# Evaluation of contemporary treatment of high- and very high-risk patients for the prevention of cardiovascular events in Europe – Methodology and rationale for the multinational observational SANTORINI study

**DOI:** 10.1016/j.athplu.2021.08.003

**Published:** 2021-08-13

**Authors:** Kausik K. Ray, Inaam Haq, Aikaterini Bilitou, Carlos Aguiar, Marcello Arca, Derek L. Connolly, Mats Eriksson, Jean Ferrières, Per Hildebrandt, Ulrich Laufs, Jose M. Mostaza, David Nanchen, Ernst Rietzschel, Timo Strandberg, Hermann Toplak, Frank L.J. Visseren, Alberico L. Catapano

**Affiliations:** a Imperial Centre for Cardiovascular Disease Prevention, Department of Primary Care and Public Health, Imperial College London, London, UK; b Daiichi Sankyo Europe, Munich, Germany; c Heart Institute, Carnaxide, Portugal; d Department of Translational and Precision Medicine, Sapienza Università di Roma, Rome, Italy; e Sandwell and West Birmingham NHS Trust, Birmingham City Hospital, Institute of Cardiovascular Sciences, University of Birmingham, Birmingham, UK; f Karolinska University Hospital, Stockholm, Sweden; g Department of Cardiology and INSERM UMR 1295, Toulouse Rangueil University Hospital, Toulouse University School of Medicine, Toulouse, France; h Frederiksberg Heart Clinic, Frederiksberg, Denmark; i University Hospital Leipzig, Leipzig, Germany; j La Paz-Carlos III Hospital, Madrid, Spain; k Center for Primary Care and Public Health (Unisanté), University of Lausanne, Lausanne, Switzerland; l Ghent University and Ghent University Hospital, Ghent, Belgium; m University of Helsinki and Helsinki University Hospital, Helsinki, Finland; n University of Oulu, Center for Life Course Health Research, Oulu, Finland; o Department of Medicine, Division of Endocrinology and Diabetology, Medical University of Graz, Graz, Austria; p Department of Vascular Medicine, University Medical Center Utrecht, Utrecht, the Netherlands; q Department of Pharmacological and Biomolecular Sciences, University of Milan and Multimedica IRCCS, Milan, Italy

**Keywords:** Cardiovascular disease, LDL cholesterol, High cardiovascular risk, Lipid-lowering therapy

## Abstract

**Background and aims:**

Clinical practice before 2019 suggests a substantial proportion of high and very high CV risk patients taking lipid-lowering therapy (LLT) would not achieve the new LDL-C goals recommended in the 2019 ESC/EAS guidelines (<70 and < 55 mg/dL, respectively). To what extent practice has changed since the last ESC/EAS guideline update is uncertain, and quantification of remaining implementation gaps may inform health policy.

**Methods:**

The SANTORINI study is a multinational, multicentre, prospective, observational, non-interventional study documenting patient data at baseline (enrolment) and at 12-month follow-up. The study recruited 9606 patients ≥18 years of age with high and very high CV risk (as assigned by the investigators) requiring LLT, with no formal patient or comparator groups. The primary objective is to document, in the real-world setting, the effectiveness of current treatment modalities in managing plasma levels of LDL-C in high- and very high-risk patients requiring LLT. Key secondary effectiveness objectives include documenting the relationship between LLT and levels of other plasma lipids, high-sensitivity C-reactive protein (hsCRP) and overall predicted CV risk over one year. Health economics and patient-relevant parameters will also be assessed.

**Conclusions:**

The SANTORINI study, which commenced after the 2019 ESC/EAS guidelines were published, is ideally placed to provide important contemporary insights into the evolving management of LLT in Europe and highlight factors contributing to the low levels of LDL-C goal achievement among high and very high CV risk patients. It is hoped the findings will help enhance patient management and reduce the burden of ASCVD in Europe.

## Highlights

• Many high and very high CV risk patients fail to achieve ESC/EAS guideline LDL-C goal.
• SANTORINI recruited 9606 high and very high CV risk patients requiring LLT.
• Real-world practice will be quantified by systematic evaluation of current LLT.
• SANTORINI will provide insights into the changing management of LLT in Europe.
• Factors contributing to low levels of LDL-C goal achievement will be highlighted.

## Introduction

Each year, cardiovascular disease (CVD) costs the EU approximately €210 billion [1] and is responsible for over 4 million deaths in Europe [2]. Low-density lipoprotein cholesterol (LDL-C) has been established as directly causal for atherosclerotic cardiovascular disease (ASCVD) [3], and due to its critical role in atherosclerotic plaque formation, lowering LDL-C is key to reducing the risk of CVD, irrespective of levels of LDL-C or drivers of future risk [2].

Lifestyle measures such as diet, exercise, and weight loss are recommended to reduce the risk of CVD; similarly, lipid-lowering therapy is also recommended in many patients at high or very high CV risk [2]. While several classes of lipid-lowering therapy (LLT) are available, decades of research have shown that statins reduce circulating levels of atherogenic lipoproteins as well as the risk of CV events in both primary and secondary prevention [2]. Furthermore, statins are available as generic therapies and thus, statins are acknowledged as the mainstay for prevention and treatment of CVD [4], and are recommended as the first line of therapy by the 2019 European Society of Cardiology (ESC)/European Atherosclerosis Society (EAS) guidelines [2]. Depending on a patient's baseline LDL-C level and the CV risk, use of high-intensity statins at the highest tolerated dose is recommended to reach LDL-C goal. If the patient does not reach the treatment goal, more intensive lowering of LDL-C with add-on therapies is recommended [2]. The aim is to mitigate the higher absolute risk by achieving lower LDL-C levels in those patients with the highest risk. The recent EU-Wide Cross-Sectional Observational Study of Lipid-Modifying Therapy Use in Secondary and Primary Care (DA VINCI study; n = 5888) showed that achievement of 2019 ESC/EAS guideline-recommended goals in very high-risk patients was 22% with high-intensity statin monotherapy compared with 58% with proprotein convertase subtilisin/kexin type 9 (PCSK9) inhibitor-based combination therapy [5]. This further supports a shift in this paradigm away from high-intensity statin therapy and towards the use of high-intensity LDL-lowering therapy based upon combination therapy, with the latter eventually becoming the proposed new standard of care [6].

Several large-scale observational studies have assessed the use of LLT in current clinical practice [5,[7], [8], [9], [10]]. These studies have clearly shown that the use of high-intensity statins and add-on therapy with ezetimibe and/or a PCSK9 inhibitor was low compared with the use of statin monotherapy. However, the new lower LDL-C goals recommended by the 2019 ESC/EAS guidelines may not always be achievable with statin monotherapy, whereas the use of add-on therapy is associated with larger reductions in LDL-C [11,12] and a higher proportion of patients achieving their LDL-C goals [5]. Specifically, the DA VINCI study demonstrated that LDL-C goal achievement was suboptimal, with fewer patients attaining the more stringent 2019 ESC/EAS guideline goals (33%) compared with the 2016 guideline goals (54%) [5,13]. Due to the rapidly changing nature of the LLT landscape, and the recent clinical recommendations for use of combination therapies, there remains a need for additional data on the effectiveness of such therapies.

The SANTORINI study aims to describe, in a real-world setting, approaches to use of LLT for the management of high- and very high-risk patients and the effectiveness of these approaches in controlling plasma levels of LDL-C. The effect of LLT on other lipid parameters will also be documented, as well as high-sensitivity C-reactive protein (hsCRP) and overall CV risk over one year, and certain health economics outcome research (HEOR) parameters.

## Patients and methods

### Patients

**Fig. 1:**
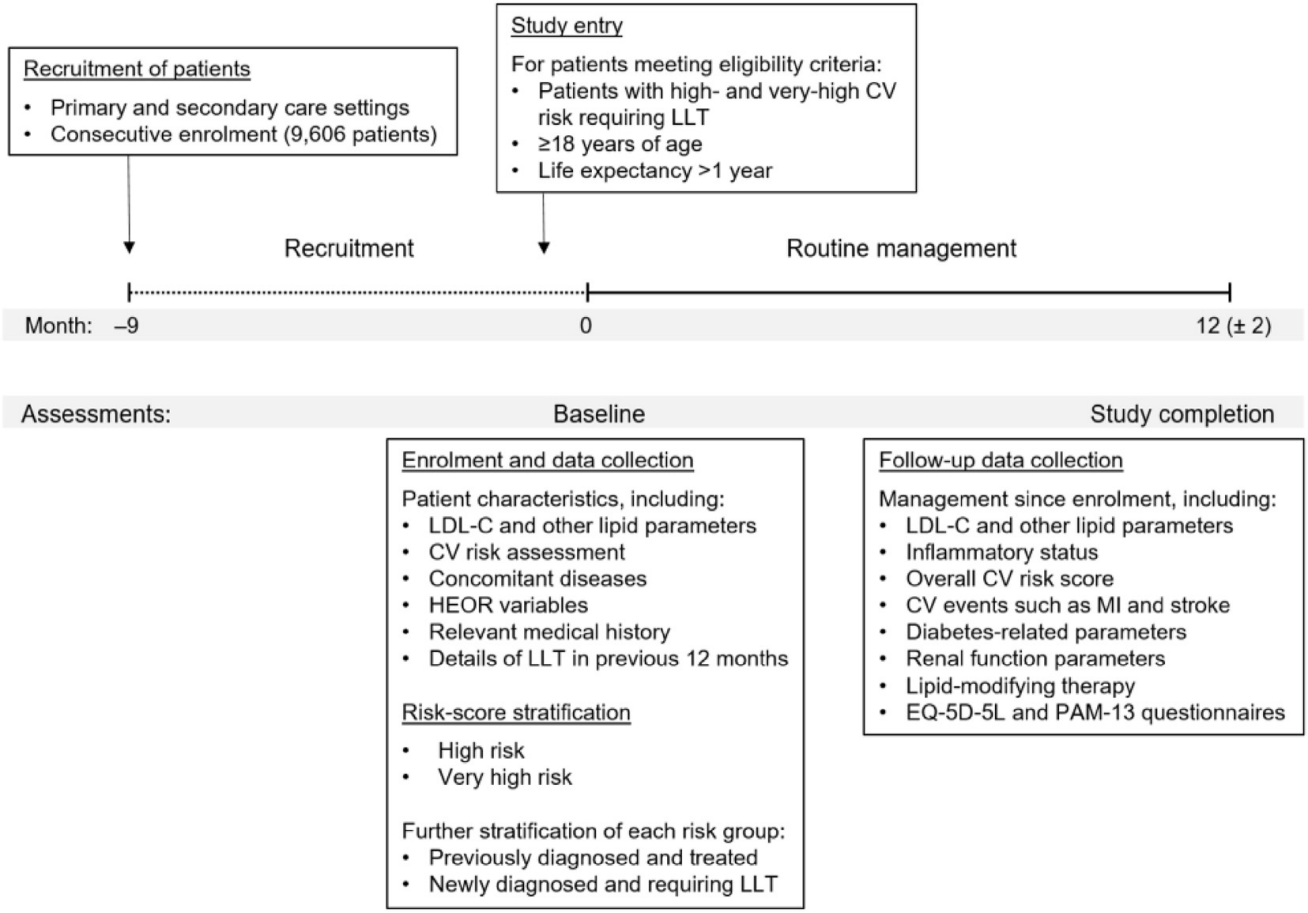
Patient enrolment and study flow. Fig. 1. CHF, congestive heart failure; CV, cardiovascular; HEOR, health economics outcome research; LDL-C, low-density lipoprotein cholesterol; LLT, lipid-lowering therapy; MI, myocardial infarction.

Patient enrolment and study flow are shown in Fig. 1.

The study enrolled 9606 high and very high CV risk patients requiring LLT from different countries and care settings (primary care and secondary care, and different specialties) consecutively from March 2020 to February 2021 at a total of 623 sites across Europe. Patients were recruited from sites in Austria, Belgium, Denmark, Finland, France, Germany, Ireland, Italy, the Netherlands, Portugal, Spain, Sweden, Switzerland and the United Kingdom.

Patients were eligible for enrolment if they were ≥18 years of age with high or very high CV risk. Risk was assigned by the investigators on enrolment, and the basis for risk category was documented. The investigator's risk categorization was the primary risk category, but risk will also be calculated during the analysis of the registry data on the basis of the documented patient information (Secondary Manifestations of ARTerial disease [SMART] [14,15], Framingham [16] and Systematic Coronary Risk Estimation [SCORE] [17] risk score systems).

Patients also had to require LLT and have an anticipated life expectancy >1 year. Patients were not to be simultaneously participating in any interventional study and had to provide written informed consent for participation. Patients will be stratified according to whether they have a high or very high CV risk status (per physicians’ assessment), as well as by whether they have previously received treatment or are newly treated. No explicit exclusion criteria are defined.

### Study design

This is a multinational, multicentre, prospective observational, non-interventional study. There are no formal patient groups planned and no comparator groups will be introduced. The patient recruitment period in each country consisted of 9 months, followed by a 12-month follow-up period per patient. Patient data was documented at baseline, at enrolment, and will be documented again at follow-up, approximately 12 ± 2 months after baseline data collection.

At the baseline data collection point, patients’ baseline characteristics and medical history were documented as well as their current LLT and any other co-medications. For the previously diagnosed and treated patients, details of LLT in the year before enrolment were also collected. This information was collected from the patients’ charts for all dyslipidemia-related visits at which the patient was seen by the physician, starting from the date of diagnosis.

At the 12-month follow-up visit, available information on patients' routine management between baseline and this data collection point will be documented. Information on efficacy and safety variables will be collected, including LDL-C and other lipid parameters, inflammatory status, and overall CV risk scores (SMART [14,15] and Framingham risk scores [16] will be calculated centrally based on collected patient information). Clinical outcomes related to CV events such as death, non-fatal myocardial infarction and stroke, and coronary revascularization, as well as diabetes related parameters, renal function parameters, and the LLT will also be collected. Previous and current use of LLT including details of dose, dose modification, switching, combination therapy, drug intolerance, insufficient responsiveness, and investigators' and patients' assessment on compliance was documented at baseline and will be documented again at annual follow-up. Patient reported outcomes, such as health-related quality of life (HRQoL) and patient satisfaction were assessed at baseline and will be assessed again at 1-year (follow-up) data collection points using the EuroQol (EQ-5D-5L) and Patient Activation Measure (PAM-13) questionnaires. The EQ-5D-5L is a generic utility measure rating the current overall health status, and the PAM-13 is a reliable and valid measure that assesses patient knowledge, skills and confidence for self-management based on 13 items. These questionnaires will be completed by the patient, preferably in the physician's office.

Electronic data capture will be used for the recording of the information. Data will be collected in standardized English electronic case report forms (eCRF). To facilitate accurate recording of data, patients can also fill in an optional memory aid (patient diary) to note important details.

As this is a non-interventional study (NCT04271280), only data that are based on routine clinical practice will be documented [18]. Treatment pattern and treatment initiation, continuation or changes are solely at the discretion of the physician and the patient. All medications will be prescribed according to usual standard of care and will not be provided by the study sponsor.

### Assessments

The primary objective is to document, in the real-world setting, the effectiveness of current treatment modalities in managing plasma levels of LDL-C in high and very high CV risk patients requiring LLT.

Key secondary objectives are to describe the relationship between treatments used as LLT and plasma levels of other lipid parameters; namely, non-high-density lipoprotein cholesterol (non-HDL-C), total cholesterol, apolipoprotein B (Apo B), triglycerides, lipoprotein (a) (Lp(a)) and HDL-C. Other key secondary effectiveness objectives are to document the relationship between treatments used as LLT and the inflammatory marker hsCRP, and patients’ overall CV risk scores.

Other secondary objectives are to describe the adverse event profile of current LLT in a real-world setting. Adverse events potentially associated with LLT include laboratory abnormalities, muscle-associated symptoms, new onset and/or worsening diabetes, changes in glycaemic status over time and adverse drug reactions associated with bempedoic acid and/or fixed dose combination with ezetimibe.

Additional secondary objectives are to document the characteristics of the sites and physicians caring for high- and very high-risk patients, and the patients’ use of LLT. This applies especially with regard to a switch from one drug class to another, a dose modification of a given drug, a switch from one statin to another, any evidence of statin intolerance or insufficient responsiveness to a statin, including a maximally tolerated statin, and any use of combination drug therapy.

Various HEOR parameters associated with the management of high- and very high-risk patients will also be assessed, encompassing patient-reported outcomes, adherence to former and current treatment, healthcare resource utilization (HCRU) and patients’ disease awareness. HCRU parameters to be assessed include hospital admissions, length of hospital stay, number of days in intensive care, and interventions. Other sociodemographic characteristics, such as insurance and employment status and education, will be collected where possible.

Parameters that will be recorded during the observation period and at 12-month follow-up are detailed in Table 1.

**Table 1:** Parameters recorded during the observation period and at follow-up.

Parameters documented at baseline (if available)	Parameters to be documented at 12-month follow-up (if available)
• Eligibility • Demographic and sociodemographic variables • Vital signs • Medical history, including CVD risk status-related medical history: o CV risk assessment with or without utilization of risk score o History/diagnosis and current status of elevated LDL-C o Heterozygous familial hypercholesterolemia (HeFH), as documented by investigator o Documented ASCVD either clinical or unequivocal on imaging^a^ • Concomitant diseases • For diabetic patients o Disease type and date of diagnosis o Treatment o Microvascular complications • Current concomitant medication for CVD, as documented by investigator • Previous/current dietary restrictions • Previous/current use of lipid-lowering treatment including details of dose, dose modification, switching, combination therapy, drug intolerance, insufficient responsiveness, investigators and patient's assessment on adherence o Statin(s) o Bile acid sequestrant(s) o Cholesterol absorption inhibitor (Ezetimibe) o PCSK9 inhibitor(s) o Nicotinic acid o Drug combinations o Other • Laboratory parameters o Lipid variables - LDL-C - HDL-C, non-HDL-C, TC, Apo B, TG, Lp(a) o Diabetes-related parameters - Fasting glucose - HbA1c o Inflammatory status - hsCRP o Renal function parameters (GFR, serum creatinine, blood urea nitrogen (BUN)) o Other laboratory parameters (liver function, serum chemistry, hematology) • HEOR parameters o Hospital admissions o Length of hospital stay o Number of days in ICU o Interventions o Other HEOR parameters o Adherence to former and current treatment o Patient reported outcomes (EQ-5D-5L^b^, PAM-13^c^)	• Vital signs • CVD risk status: o CV risk assessment with or without utilization of risk score o History/diagnosis and current status of elevated LDL-C o Heterozygous familial hypercholesterolemia (HeFH) o Documented ASCVD either clinical or unequivocal on imaging • Concomitant diseases o Pre-specified and other CVD newly diagnosed/worsened from baseline o Time to disease onset/first event o Pre-specified and other symptomatic CVD newly diagnosed/symptomatic since baseline o Time to disease stopped being symptomatic o Pre-specified CV procedures since baseline o Time to last CV procedure • Deaths o Time to death and cause of death • For diabetic patients o Disease type and date of diagnosis o Treatment o Microvascular complications • Current concomitant medication for CVD • Previous/current dietary restrictions • Previous/current use of lipid-lowering treatment including details of dose, dose modification, switching, combination therapy, drug intolerance, insufficient responsiveness, investigators and patient's assessment on adherence o Statin(s) o Bile acid sequestrant(s) o Cholesterol absorption inhibitor (Ezetimibe) o PCSK9 inhibitor(s) o Nicotinic acid o Drug combinations o Other • Clinical events associated with the lipid-lowering therapies: o Insufficient lipid lowering efficacy o Muscle-associated symptoms (taken from patient chart) o New-onset diabetes mellitus o Drug-drug interaction o Laboratory abnormalities o Other • Neurocognitive impairment • Non-adherence to lipid-lowering therapies, as assessed by investigator • Laboratory parameters, as measured and provided by individual study sites o Lipid variables - LDL-C - HDL-C, non-HDL-C, TC, Apo B, TG, Lp(a) o Diabetes-related parameters - Fasting glucose - HbA1c o Inflammatory status - hsCRP o Renal function parameters (GFR, serum creatinine, blood urea nitrogen (BUN)) o Other laboratory parameters (liver function, serum chemistry, hematology) • HEOR parameters o Hospital admissions o Length of hospital stay o Number of days in ICU o Interventions o Other HEOR parameters (e.g. insurance status, employment status, education) o Patient reported outcomes (EQ-5D-5L^b^, PAM-13^c^)

ACS, acute coronary syndrome; Apo B, apolipoprotein B; ASCVD, atherosclerotic cardiovascular disease; BUN, blood urea nitrogen; CABG, coronary artery bypass graft surgery; CT, computed tomography; CV, cardiovascular; GFR, glomerular filtration rate; HDL-C, high-density lipoprotein cholesterol; HEOR, health economics outcome research; hsCRP, high-sensitivity C-reactive protein; ICU, intensive care unit; Lp(a), lipoprotein (a); MI, myocardial infarction; PCI, percutaneous coronary intervention; PCSK9, proprotein convertase subtilisin/kexin type 9; TC, total cholesterol; TG, triglycerides; TIA, transient ischaemic attack.

a Documented ASCVD includes previous ACS (MI or unstable angina), stable angina, coronary revascularization (PCI, CABG, and other arterial revascularization procedures), stroke and TIA, and peripheral arterial disease. Unequivocally documented ASCVD on imaging includes those findings that are known to be predictive of clinical events, such as significant plaque on coronary angiography or CT scan (multivessel coronary disease with two major epicardial arteries having >50% stenosis), or on carotid ultrasound [2].

b The EuroQol (EQ-5D-5L) consists of five domains (mobility, self-care, usual activities, pain or discomfort, and anxiety or depression) and a visual analogue scale (VAS). The scores range from 0 to 100 based on the level of health for each domain given by participants. A score of 100 indicates current health is equivalent to full health; a score of 0 indicates that the current health is equivalent to death. According to the scores of the five domains, a sum utility score is calculated ranging from 0 to 1. A score of 1 represents a perfect state. In addition, patients rate their current health on a 20-cm vertical VAS scored from 0 to 100 reflecting the continuum from a best imaginable to the worst imaginable health state.

c PAM-13 assesses patient knowledge, skills, and confidence for self-management based on 13 items. Individuals are segmented into one of four activation levels along an empirically derived 100-point scale. Each level provides insight into an extensive array of health-related characteristics, including attitudes, motivators, and behaviors. Individuals in the lowest activation level do not yet understand the importance of their role in managing their own health, and have significant knowledge gaps and limited self-management skills. Individuals in the highest activation level are proactive with their health, have developed strong self-management skills, and are resilient in times of stress or change.

### Sample size considerations

As this registry is intended to collect data from actual clinical practice and the statistical analysis will be performed in a purely exploratory and descriptive way, no primary parameter has been defined for the sample size calculation. On a pragmatic basis, a sample size of 8000 patients from approximately 800 sites was deemed necessary to provide sufficient precision (measured by width of 95% confidence intervals [CI]) for estimation of the rates of CV death, non-fatal myocardial infarction, non-fatal stroke, and coronary revascularization during one-year follow-up.

### Statistical analysis

Three populations will be defined: i) the All-Documented Patient Set (APS), which consists of all patients with any eCRF documentation; ii) patients with adequately completed recruitment information (Baseline Analysis Set [BAS] population); iii) patients having completed 1-year follow-up (Full Analysis Set [FAS] population).

LDL-C values at baseline and 1-year follow-up will be analyzed descriptively as a continuous parameter and as LDL-C categories. Absolute and percentage change from baseline at 1-year follow-up in LDL-C will be analyzed. Absolute and percentage change from baseline at 1-year follow-up will also be analyzed for the other lipid parameters, hsCRP, and overall CV risk assessment. The CV risk assessment will include categorical assessment for all patients, continuous and categorical assessment for SMART and Framingham risk score as calculated by the investigator as well as centrally, and risk score ratios as surrogate relative risks; for each patient, a ratio of baseline and 1-year SMART or Framingham risk score will be calculated, and summary statistics of the ratios will be calculated.

Baseline parameters such as risk classification by investigator, central risk categorization, previously or newly treated, class of LLT, age, sex, and region will be considered for subgroup analysis. This will be applied for the primary and secondary effectiveness parameters, for primary and secondary laboratory parameters values at baseline and 1-year follow-up and for all analyses of risk scores.

Analysis of changes from baseline in primary and secondary effectiveness parameters will be performed by paired Wilcoxon signed rank test and paired *t*-test. Shapiro-Wilk test results and Q-Q plots will be provided for the assessment of normality. Comparison of changes from baseline for the primary and secondary effectiveness parameters between subgroups will be done by Kruskal-Wallis test and ANOVA. Shapiro-Wilk test results and Q-Q plots will be provided for the assessment of normality in each subgroup and Levene's test results will be provided for the assessment of homogeneity of variance. Adjusted analyses for age, sex and country effects will also be performed for the parametric approach by means of a multivariate ANOVA. Time-to-event variables will be analyzed via a Cox proportional hazard regression model presenting hazard ratios and the corresponding 95% CIs. Additionally, Kaplan-Meier curves will be presented for time-to-event variables.

All statistical tests will be two sided and will be performed at the 5% level of significance, unless otherwise stated. Two-sided 95% CI will be presented for LDL-C goal (overall and for high- and very high-risk patients), but should be interpreted in an exploratory descriptive way. No primary endpoint was defined in this study. No formal statistical tests will be performed within the statistical analysis. Any p-value given should be interpreted in a purely exploratory sense.

### Ethics approval and consent to participate

The SANTORINI study will be performed in accordance with the Declaration of Helsinki and Good Clinical Practice. All patients will be asked to provide written informed consent before participating in the study.

## Discussion

Treatment guidelines, which are based to a considerable extent on the outcomes of randomized clinical trials (RCTs), offer health care providers advice and direction with respect to the management of patients with high and very high CV risk. The updated 2019 ESC/EAS guidelines recommend LDL-C reductions of >50% from baseline and an LDL-C goal of <55 mg/dL in very high-risk patients. Furthermore, for patients who experience a recurrent vascular event within 2 years while taking maximally tolerated statin therapy, an LDL-C goal of <40 mg/dL can be considered. For high-risk patients, an LDL-C reduction of >50% from baseline and an LDL-C goal of <70 mg/dL are recommended. However, RCTs are conducted within a strictly controlled environment. Achieving these LDL-C goals outside of clinical trials is challenging and, therefore, there is an important need to understand the situation in actual clinical practice, and how patients can be helped to achieve their LDL-C goals.

Since the DA VINCI study provided fresh data on LLT usage and LDL-C goal achievement, the LLT landscape has changed, with ezetimibe becoming more widely available as a generic and monoclonal antibody prices having fallen. The ways in which this may have affected usage of these drugs as well as other LLT is not yet known. Thus, SANTORINI aims to describe approaches to use of LLT and the effectiveness of current medical practice for the management of high- and very high-risk patients. A better understanding of the use of LLT to mitigate against elevated CV risk across European countries may help enhance patient management and reduce the burden of ASCVD in Europe.

Despite the widespread availability and use of statins, and the availability of other drug classes, including older and newer agents (such as bile acid sequestrants, cholesterol absorption inhibitors, PCSK9 inhibitors, nicotinic acid, and their various combinations), a substantial proportion of high- and very high-risk patients are not at goal [7]. According to various European observational studies, about 70–80% do not reach their LDL-C target [5,7,9,10,[19], [20], [21]]. Of note, the large global Dyslipidemia International Study (DYSIS), in which the majority of patients were treated in a primary care setting, showed that only 21.7% of very high-risk patients attained their LDL-C goal [22]. The DYSIS II study focused on very high-risk patients and showed that, in patients with stable coronary heart disease (CHD), 29.6% of patients treated with LLT attained an LDL-C level of <70 mg/dL compared with 8.3% of those not being treated (p < 0.0001) [9]. In patients hospitalized for an acute coronary syndrome (ACS) event, these values were 23.2% and 2.9%, respectively (p < 0.0001). The European Action on Secondary and Primary Prevention by Intervention to Reduce Events (EUROASPIRE) IV cross-sectional survey also evaluated risk factor management in primary care [23]. In patients without a history of ASCVD, 32.7% of those prescribed LLT reached an LDL-C target of <100 mg/dL. Just 10.7% of patients without any LLT had an LDL-C below this level. In a separate analysis of EUROASPIRE IV in patients with a diagnosis of first or recurrent CHD, 19.3% of such patients achieved an LDL-C level of <70 mg/dL; attainment of this target LDL-C value was 26.6% among patients on high-intensity statins [24]. The DA VINCI study showed that overall, a vast majority of LLT in Europe was monotherapy – primarily moderate-intensity statin (51.8%) and high-intensity statin (27.6%) – and that only 33% of patients attained the 2019 ESC/EAS guideline LDL-C goals [5]. In the very high-risk population, 18% achieved LDL-C goals of <55 mg/dL; goal achievement was 14%, 16% and 22% for patients receiving low-, moderate- and high-intensity statin monotherapy, respectively. Overall, combination therapy was associated with a higher rate of goal achievement than statin monotherapy, with 37% of those receiving ezetimibe combination therapy and 57% who received PCSK9 inhibitor combination therapy reaching risk-based LDL-C goals [5].

However, many of these studies have been conducted in large hospitals or university centres, which raises questions about how representative or applicable the results may be to general practice and the wider population. The SANTORINI study will be comparatively larger, specifically focuses on the highest CV risk categories, and has a longitudinal follow-up design. This will provide important contemporary real-world insights into the current utilization of LLT in clinical practice since the release of the 2019 ESC/EAS guidelines. This is especially important for patients who are at risk of, or have already had, a CV event, and who may be undertreated and/or unable to reach guideline recommended goals. Furthermore, none of the previous studies have assessed as wide a selection of parameters as those in SANTORINI. The study benefits from the inclusion of lipid parameters other than LDL-C, such as Apo B and Lp(a), and the inflammatory biomarker, hsCRP, as secondary endpoints – although hsCRP alone may not be enough to determine overall changes in vascular inflammation without other inflammatory markers to aid interpretation. The assessment of overall CV risk, characteristics of the sites and the specialties of the physicians, and additional safety and HEOR outcomes also provides this study with additional value. Moreover, as patients’ journeys seem to vary between different countries, by documenting meaningful patient-related parameters the SANTORINI study can be expected to provide important insights into the care process, country-by-country. The study could also help assess the different use of specific LLTs in different countries.

The observational design of the study means that only data from routine clinical treatment can be obtained. Therefore, although this study will assess ‘real-world’ management of high- and very high-risk patients, it should be noted that the study population represents a selected group of patients that have been referred to specifically chosen lipid experts. Thus, the data on LDL-C and treatment approaches should be viewed as a ‘best case scenario’. To encourage participation in the study and truly capture the difference in clinical practice, the observational plan did not specify standardized lipid measurement. Therefore, there will be inherent variances, for example in laboratory tests and calculated LDL-C, which will introduce a degree of variability to the results. Patient memory aids and medical records at the site will be used to support the precise recording of the time between the two annual data documentation time points, and the data collected will be considered representative of the whole study population. Differences between the countries/regions might also occur, especially when rating adherence, and there may also be under-representation in the trial from some smaller regions. Therefore, the interpretation should consider regional differences.

In conclusion, SANTORINI will document the current management of high and very high CV risk patients in Europe, including medical outcomes and those relevant to HEOR. This will contribute to an understanding of the management of such patients and, ultimately, help to characterize the clinical and economic burden associated with the management of ASCVD in Europe. The study will also help to provide important insights into factors contributing to the poor levels of LDL-C goal achievement among those patients with high and very high CV risk across Europe.

## Conflicts of interest/Competing interests

KR has received lecture fees from Aegerion Pharmaceuticals, Kowa, Cipla, Algorithm, and Zuelling Pharma; grant support, paid to his institution, lecture fees, and advisory board fees from Amgen, Regeneron Pharmaceuticals/Sanofi, Daiichi Sankyo and Pfizer; lecture fees and fees for serving on steering committees for trials from AstraZeneca and Eli Lilly; fees for serving on steering committees for trials from Cerenis Therapeutics, the Medicines Company, and Esperion; advisory board fees from New Amsterdam Pharma, Akcea Therapeutics, Novartis, Silence Therapeutics, Bayer, and Daiichi Sankyo; lecture fees and advisory board fees from Takeda, Boehringer Ingelheim, and Dr. Reddy's Laboratories; consulting fees from Silence Therapeutics and Bayer; grant support and advisory board fees from Merck Sharp & Dohme; fees for serving on a clinical events adjudication committee from AbbVie; and fees for serving as principal investigator for a trial from Resverlogix. JF has received lecture fees from Amarin, Amgen, Lilly, Mylan, Sanofi and Servier. CA has received honoraria for consultancy and/or lectures fees from Abbott, Amgen, BIAL, Daiichi Sankyo, Merck Sharp & Dohme, Mylan, Servier and Tecnimede. DC has received fees for advisory boards, research and lectures from Amgen, Bayer, BMS, Boehringer Ingelheim, Daiichi Sankyo, Novartis, Pfizer and Sanofi. TS has received consulting fees and research and educational grants from Amgen, Novartis, Orion Pharma, Pfizer, Sankyo, Sanofi and Servier. HT has received lecture fees from Amgen, Daiichi Sankyo, Mylan, MSD, Novonordisk, Novartis, Pfizer and Sanofi, as well as research grants from Amgen, Daiichi Sankyo, Novartis, Novonordisk and Sanofi. MA has received research grant support and lecturing fees from Amgen, Amryt, IONIS/Akcea Therapeutics, Daiichi Sankyo, Novartis, Pfizer, Regeneron and Sanofi. ER has received unrestricted educational grants from Amgen, Merck Sharp & Dohme, AstraZeneca, Sanofi and Unilever, speakers' or consultancy fees from Daiichi Sankyo, Novonordisk, Boehringer Ingelheim, Amgen, Sanofi, Novartis and Teva; all paid directly to Ghent University. DN is or has been an investigator in clinical studies sponsored by Amgen, Pfizer, Daiichi Sankyo and Novartis; he has not received any personal fees for this work. JMM has received lecture and advisory board fees from Amgen, Daiichi Sankyo, Ferrer, Novartis, Pfizer and Sanofi. UL has received honoraria for lectures or consulting from Amgen, Daiichi Sankyo, Novartis and Sanofi. ME has received lecture and advisory board fees from Sanofi, and Amgen, advisory board fees from Akcea Therapeutics, and consulting fees from Novartis. IH and AB are employees of Daiichi Sankyo. FV and PH declare no conflicts of interest.

## Funding

This study is funded by Daiichi Sankyo Europe GmbH, Munich, Germany, who defined the study design, data collection and analysis. Six contract research organizations were contracted to conduct the study in 14 countries on behalf of Daiichi Sankyo Europe.

## Authors' contributions

All authors reviewed and edited the manuscript and approved the final version. In addition, KR, AC, IH and AB contributed to study conceptualization and methodology. All other authors contributed to the study investigation.

## Availability of data and material

De-identified individual participant data and applicable supporting clinical study documents are available on request, depending on circumstances, at https://vivli.org. In cases in which clinical study data and supporting documents are provided pursuant to the sponsor's policies and procedures, the sponsor will continue to protect the privacy of the clinical study participants. Details on data sharing criteria and the procedure for requesting access can be found at https://vivli.org/ourmember/daiichi-sankyo/.

## Ethics approval

The SANTORINI study will be performed in accordance with the Declaration of Helsinki and Good Clinical Practice.

## Consent to participate

All patients were asked to provide written informed consent before participating in the study.

## Declaration of competing interest

The authors declare the following financial interests/personal relationships which may be considered as potential competing interests:
